# Premenstrual syndrome, coping mechanisms and associated factors among Wolkite university female regular students, Ethiopia, 2021

**DOI:** 10.1186/s12905-022-01658-5

**Published:** 2022-03-23

**Authors:** Natnael Eshetu, Haimanot Abebe, Elishaday Fikadu, Serkalem Getaye, Seid Jemal, Shegaw Geze, Yibeltal Mesfin, Seboka Abebe, Daniel Tsega, Bitew Tefera, Wubishet Tesfaye

**Affiliations:** 1 Department of Midwifery, Wolkite University, Wolkite, Ethiopia; 2 Department of Nursing, Wolkite University, Wolkite, Ethiopia

## Abstract

**Background:**

Premenstrual syndrome (PMS) is used to describe physical, cognitive, affective, and behavioral symptoms that affect young and middle-aged women occurring cyclically during the luteal phase of the menstrual cycle. Despite the considerable prevalence and impact of PMS on individuals, their families and communities that interferes with the development of nations, many professionals are still unaware of it and little attention has been given in developing countries like Ethiopia, especially for university students. Therefore, this study was aimed for assessing the magnitude of premenstrual syndrome, associated factors and coping mechanisms among Wolkite university female regular students, 2021.

**Methods:**

Institutional-based cross sectional study was conducted among Wolkite University regular female students from June 15/10/2021 to 30/10/2021 by using simple random and multistage with systematic random sampling technique to select the study participants (n = 591). Data were collected using a self-administered, pre-tested, semi-structured questionnaire. Premenstrual syndrome scales comprised of 40 questions with three sub-scales were used to determine Premenstrual syndrome. Data were cleaned, coded and entered into Epi-data version-3.1, and analyzed using SPSS software version 25. Descriptive statistics were computed for independent variables as well as for coping mechanisms and presented in narration, tables and graphs. Analytic analysis schemes including bi-variable and multivariable logistic regression were computed to identify factors associated with premenstrual syndrome and those variables with a *P *value of < 0.05 in multivariable analysis were declared as statistically significant.

**Result:**

From the total of 631 study subjects, only 591 had completed the questionnaire, giving a response rate of 93.7%. From 591 study participants, 224 (37.9%) [95% CI: (34, 40.9)] of them had premenstrual syndrome. Abdominal cramp (78.8%), depression (73.3%) and fatigue (72.9%) were frequent premenstrual symptoms experienced by students. Having family history of PMS [AOR: 4.05; 95% CI: (2.49, 6.58)], no history of sexual intercourse [AOR: 2; 95% CI: (1.12, 3.47)], severe menstrual pain intensity [AOR: 3.09; 95% CI: (1.58, 6.05)], irregular menstrual cycle [AOR: 2.26; 95% CI: (1.41, 3.62)], early age of menarche (< 13 years) [AOR: 2.64; 95% CI: (1.34, 5.19)], long duration of menses (≥ 7 days) [AOR: 3.56; 95% CI: (1.53, 8.37)] and using many pads (> 8) during menstruation [AOR: 4.44; 95% CI: (2.16, 9.12)] were factors significantly associated with premenstrual syndrome. 93.4% of students apply at least one coping mechanism for premenstrual symptoms, of which; taking rest (67.6%) and sleeping (60.7%) were common strategies.

**Conclusion:**

In this study, premenstrual syndrome was found to be a problem of many students. Abdominal cramp, depressed feeling and fatigue were the predominant premenstrual symptoms experienced by students. Taking rest and sleeping were mostly applied by students as a coping mechanism. Family history of PMS, no history of sexual intercourse, intense menstrual pain, use of many pads during menstruation, irregular menstrual cycle, early menarche, and long duration of menses were found to be predictors of premenstrual syndrome. PMS needs great attention as part of the health care service in Ethiopia by involving all stockholders, including policy makers and health care professionals, to reduce its impact on the academic performance of university students.

**Supplementary Information:**

The online version contains supplementary material available at 10.1186/s12905-022-01658-5.

## Background

Menstruation is a cyclic physiologic process and natural part of a woman’s life characterized by the flow of blood and endometrium from the uterine cavity [1]. This cyclic hormonal functioning is usually accompanied by changes in several physical and psychological aspects, which is described as premenstrual syndrome [2, 3]. Premenstrual syndrome (PMS) is defined as a collection of recurrent physical, cognitive, affective, and behavioral symptoms affecting women, occurring cyclically during the luteal phase of the menstrual cycle and resolving at or within a few days of the onset of menstruation [4]. Even if many women experience PMS symptoms, they do not perceive these symptoms as either distressing or debilitating [5]. The definite etiology of PMS is not well understood and may be complex and multifactorial. Change in level of ovarian steroid hormones, decrease in endogenous opioid activity during the late luteal phase of the cycle and genetics may play a role in the occurrence of PMS [6]. There are no specific laboratory tests and physical examination findings to confirm PMS but a symptom calendar can help to identify the troublesome symptoms[7].

Premenstrual syndrome for most women starts at the age of menarche. Many symptoms have been known to occur in PMS but the most frequently occurring symptoms include headache, fatigue, bloating, backache, breast tenderness, food cravings, fatigue, anxiety, irritability, social withdrawal and depression [8]. Physiological and psychological changes in PMS vary dramatically in severity and between individuals. Slight symptoms during the premenstrual period which do not interfere in the daily activities of women are not considered to form part of the diagnosis of PMS [9]. The more severe form of PMS is known as premenstrual dysphoric disorder (PMDD) and 3–8% of women may experience it [10].

Premenstrual syndrome is a public health problem that affects more than half of reproductive age women globally, including in Ethiopia [3] and studies have shown the prevalence of PMS varies from place to place and ranges between 5 and 90% [11]. Previous studies showed that it prevalence was 7.1% in France University Hospital in Beirut [12], Switzerland (91%) [13], Iran (30.7%) [10], Saudi Arabia (35.6%) [14], Sistan and Baluchestan University (85.6%) [9], Gujarat (18.4%) [15], Thai (16.8%) [16], Bahir Dar University, Ethiopia (72.8%) [1], Debremarkos town, Ethiopia (81.3%) [8], Mekelle University (83.2%) [4] and Addis ketema preparatory school, Ethiopia was 86.1%) [17].

Many menstruating women usually complain about symptoms of premenstrual syndrome that affects their interpersonal relations or interferes with normal activities [10]. Even if there is no previous study at Wolkite university, some studies conducted elsewhere globally showed that PMS is associated with class absenteeism, withdrawal, poor quality of life and poor academic performance of university students[18].

Premenstrual symptoms are experienced by up to 90% of all women of reproductive age, which represents a significant public health problem in young girls globally [7, 14, 18]. Unlike other psychophysical conditions that affect women on a daily basis, the burden of PMS may be misperceived as less severe because it affects only a subset of women during their luteal cycle phase[19]. This huge burden is strongly associated with poor physical health, poor quality of life and psychological distress [13], that interferes with work productivity, home or daily activities, social activities, and interference with relationships [16].

PMS affecting menstruating women continues until menopause and is considered as a chronic illness similar to diabetic mellitus, hypertension and others. It has a great burden on women’s health-related quality of life, which interferes with the development of nations by decreasing daily activity [4, 20]. PMS affects not only the woman, but also the family, society and the nation at large. It is associated with work productivity impairment, school and work absenteeism, thus, posing a potential economic burden for the society [6]. It has great financial burden manifested by frequent health care visits, drug expense and loss of work days [19] and also disturbs friend, family and sexual relationships [5].

Premenstrual syndrome is an additional source of stress for female university students that affects their academic level of performance [4, 12, 17]. Insomnia and emotional disturbance as a premenstrual syndrome affects the mental health and work performance of university students, leading to class absenteeism and committing of work-related mistakes [12]. Its burden is significant that it can disrupt relationships with other students and studying roles [6, 16], associated with increased health care costs, frequently missing classes and exams, scoring a lower grade and even school [8, 19].

Previously identified factors associated with premenstrual syndrome include; advanced reproductive age, single marital status, rural residency, lower educational status, unemployment, positive family history, and earlier age of menarche, irregular menstrual cycle and smoking [1, 7, 8, 13, 14]. Even though PMS is a condition seriously affecting a woman’s life, it has been reported that only 59.6% of women with PMS symptoms would like treatment for their complaints and 28.8% seek medical help and are also reluctant to seek help for treatable PMS symptoms because of attitudinal barriers regardless of the severity of their PMS symptoms [11]. Even if there are previously identified effective coping mechanisms for PMS like; regular exercise [17], taking rest [21], massage [22], listening to music [11], hot drink[23], taking analgesics [4] and taking a hot shower, most women consider it as normal physiological process that nothing should be done [6, 14].

Generally, PMS is a wide-spread public health problem affecting more than half of world reproductive age women, including university students associated with poor quality of life and productivity, but little is known about contributing factors and effective coping mechanisms. Despite the considerable prevalence of PMS, many professionals are still unaware of its impact on individuals, their families and communities and little or no attention has been given by the government bodies specifically in developing countries like Ethiopia [8, 13]. Since there are only a few studies in Ethiopia in general and among university students in particulars with great discrepancy on its magnitude and contributing factors, which makes it difficult to generalize, scientific evidence is needed at Wolkite University on which all interventions should be based. Therefore, this study was aimed to assess the magnitude of PMS, coping mechanisms and factors associated among Wolkite University students, which helps to avert the problem by providing means of avoiding modifiable factors and applying effective coping strategies for non-modifiable factors to reduce its effect on students’ academic performance.

## Methods

### Study design, setting and sampling

An institutional based cross sectional study was conducted from June 15 to 30/10/2021G.C at Wolkite University, which is located in Wolkite town, Gurage zone, South Nations Nationalities and people region of Ethiopia. Wolkite University is one of Ethiopia governmental higher educational institutions established in 2004 E.C, which is located 158 km away from Addis Ababa, the capital city of Ethiopia, and 135 km from Hawassa, the capital city of Sidama regional state and educating students in different fields of specializations since its establishment.

Wolkite University regular female students in the 2021 academic year were included in this study, whereas, those regular female students who were critically ill during the data collection period and those who did not start menstruation were excluded. The minimum required sample size for this study was calculated using both a single and double population proportion formula by taking the prevalence of premenstrual syndrome as 53% based on a study conducted in western Ethiopia [26], at 95% CI by assuming a margin of error 5% = 0.05), 90% power, 1:1 ratio of exposed to non-exposed outcomes, multiplied by 1.5 for design effect and adding 10% for possible non response rate and taking the maximum value the final sample was 631 based on the above assumptions. Simple random and multistage with systematic random sampling techniques were used to select the study participants. Wolkite University educates 5,960 (1704 were female) students in regular program in 9 faculties, namely: Medicine and health science (210), Engineering technology (505), Natural and computational science (172), Business and Economics (145), Computer science (170), Behavioral science (114), Agriculture (145), Law (96) and Social Science College (147). From 9 faculties, 3 was selected by using the lottery method and by using student identity number obtained registrar office as a sampling frame, systematic random sampling technique (K = 2) with proportional allocation to each selected faculty was used to select the study participants (see Additional file 1).

### Operational definition

#### Premenstrual syndrome (PMS)

Participants who scored 80 and above based on a premenstrual syndrome scale consisting of symptoms occurring about seven days prior to menstrual bleeding and ending about the time the bleeding starts[21]. The increase in the scores reflects the PMS severity. Based on the percentage of scores—“no symptoms” (1–40), “mild” (41–80), “moderate” (81–120), “severe” (121–160) and “very severe” (161–200) [21]. Coping mechanism: Participants report of any action taken attempting to alleviate or decrease premenstrual symptoms regardless of its severity [24].

### Data collection tool and procedure

A pre-tested semi-structured self-administered questionnaire was used to collect data. The questionnaire was adapted from a previous studies [25] and reviewing of relevant literatures to the problem under study. The questionnaire included socio-demographic information, life style and behavioural factors, reproductive and menustral related variables and coping mechanisms for PMS gathered from different literatures. The premenstrual syndrome scale (PMSS) comprised of 40 questions with three sub-scales namely, physiological, psychological and behavioural symptoms was used to assess the presence of PMS. The measurements was follow scoring system as “never” was scored as “1”, rarely as “2”, sometimes as “3”, very often as “4” and always as “5” points and scores of 80 and above indicates the occurrence of PMS [25]. For coping mechanisms, students who experienced any premenstrual symptoms were asked, using open-ended questions, what attempts they make to cope with these symptoms and these responses were subsequently grouped within categories.

Six data collectors and three supervisors were involved in the data collection process (two data collectors for each faculty) after one day training was given by the principal investigators. The questionnaire was distributed to each selected study participants during class session and at dormitory. After informed consent was obtained from each participant they were allowed to complete the questionnaire and any ambiguity during data collection was clarified by each assigned data collector.

### Data quality control

The quality of the data was assured through using carefully design standard data collection tool, translation and retranslation and 5% pretesting of the questionnaire before the actual data collection. All completed questionnaire was examined for its completeness and consistency during data collection time and at the end of each day. Cronbach's alpha value was used to check the reliability of the questionnaire which accounted for 0.86 and its quality was assured through experts’ evaluation. Data were entered directly in to Epi-data software and cleaned and checked for completeness and accuracy before analysis.

### Data processing and analysis

Data was entered using Epi-data version 3.1 and analyzed using the SPSS database program version 25. After cleaning the data, frequencies and percentages was calculated to all variables which are related to the objectives of the study and presented in tables, text and graphs. Data was analyzed to identify factors associated with premenstrual syndrome using standard binary logistic regression analysis. Odds ratio with 95% confidence interval was computed to assess the presence and degree of association between dependent and independent variables. Variables with *P *value < 0.05 in bi-variable logistic regression analysis were included in multi-variable analysis and those factors with *P *value < 0.05 at 95% confidence interval in multi-variable analysis were considered as statistically significant. Moreover, variance inflation factor (VIF) and tolerance to check for multi-collinarity, and Hosmer and Lemeshow goodness of fit test to check for model fitness was used.

## Result

### Socio-demographic characteristics

**Fig. 1:**
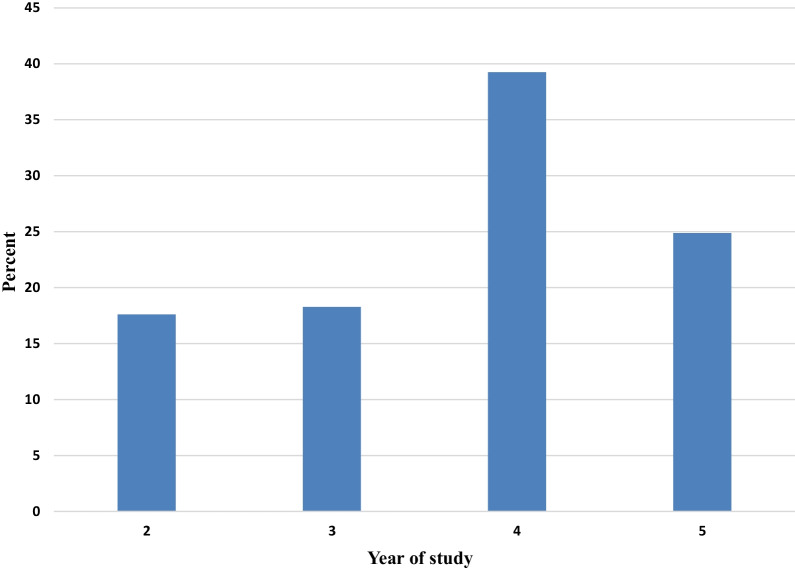
Percentage distribution of study participants in terms of year of study, Wolkite University, Wolkite, Ethiopia, 2021

From the total 631 study subjects, only 591 had completed the questionnaire, this makes the response rate 93.7%. The mean age of the participants was 21.5 years ± 1.7 standard deviation ranging from 17–26 and majority of them 429 (72.6) were coming from urban areas, following orthodox religion 334 (56.5) (see Additional File 2). Most of the study participants were also 4^th^ year student (Fig. 1).

### Life style and behavioral factors

**Fig. 2:**
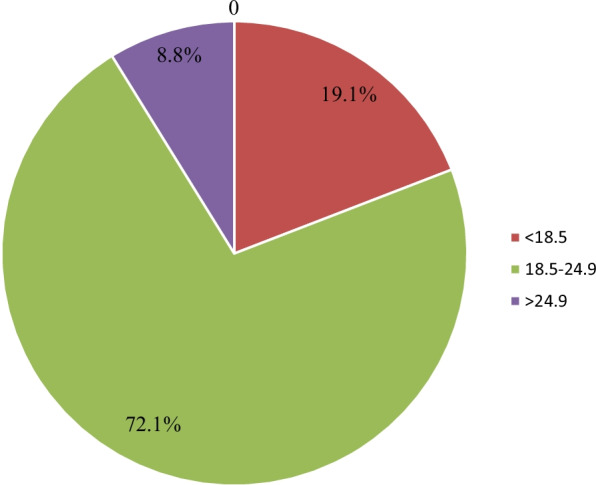
Body mass index (BMI) (Wt./Ht.^2^) category of Wolkite University regular female students, Wolkite, Ethiopia, 2021

From 591 study participants, above half of them 408 (69%) not drink alcohol at all and majority of them were non-smokers 554 (93.7%). Only 23 (3.9%) of them had habit of regular exercise and above half of them 407 (68.9%) had no history of sexual intercourse and nearly 2% of them had chronic disease, of which HIV/AIDS accounts 45.5% (Table 1). Majority of the study participants had body mass index (BMI) ranging from 18.5 kg/m^2^-24.9 kg/m^2^. (Fig. 2).

**Table 1:** Life style and behavioral characteristics of study participants, Wolkite University, Wolkite, Ethiopia, 2021

Life style and behavioral factors	Category	Frequency	Percentage (%)
Drinking alcohol	Never	408	69.1
	Sometimes	168	28.4
	Always	15	2.5
Smoking cigarrete	Never smoke	554	93.7
	Current smoker	22	3.7
	Ex-smoker	15	2.5
Chewing chat	Never	542	91.7
	Occasionally	43	7.3
	Regularily	6	1
Habit of regular exercise	No	329	55.7
	Occasionally	239	40.4
	Regularly	23	3.9
History of sexual intercourse	No	407	68.9
	Yes	184	31.1
Daily sugar consumption	< 6 tea spoon	435	73.6
	6–12 tea spoon	128	21.7
	> 12 tea spoon	28	4.7
Do you drink coffee daily?	No	342	57.9
	Yes	249	42.1
Do you drink tea daily?	Yes	534	90.4
	No	57	9.6
Do you drink milk?	Sometimes	395	66.8
	No	146	24.7
	At least one glass per day	50	8.5

### Reproductive and menstrual related characteristics

In this study above half of the participants 390 (66%) had information about premenstrual syndromes and their mean age at menarche was 14.2 years ± 1.5 standard deviation with a mean menstrual duration of 4.6 days. Among participants, 133 (22.5%) had perceived their menstrual pain intensity level as sever and 394(66.7%) of female students had regular menstrual cycle. (Table 2).

**Table 2:** Reproductive and menstrual related characteristics of study participants, Wolkite University, Ethiopia, 2021

Reproductive and menstrual related factors	Category	Frequency	Percentage (%)
Do you have information about PMS?	Yes	390	66
	No	201	34
Age at menarche	< 13 years	65	11
	> 13 years	526	89
Menstrual bleeding duration	1–3 days	137	23.2
	4–6 days	396	67
	> 7 days	58	9.8
Menstrual pain intensity	Mild	145	24.5
	Moderate	313	53
	Severe	133	22.5
Menstrual interval in days	< 21 days	123	20.8
	21–35 days	409	69.2
	> 35 days	59	10
Number of pads (modes) used during menstruation	< 4 pads	116	19.6
	4–8 pads	326	55.2
	> 8 pads	149	25.2
Perceived amount of menstrual bleeding	Mild	77	13
	Moderate	398	67.3
	Heavy	100	16.9
	Extremely heavy	16	2.7
Do your menstrual cycle regular	Yes	394	66.7
	No	197	33.3
History of pregnancy	No	531	89.8
	Yes	60	10.2
Use of any hormonal contraceptives	No	455	77
	Yes	136	23

### Premenstrual symptoms

Premenstrual symptoms experienced by students were also investigated. Among 591 study participants, most reported physiological symptoms were abdominal cramps 466 (78.8%), fatigue 431 (72.9%), psychological symptoms like depression 433 (73.3%) and mood swings 415 (70.2%), and frequent behavioral symptoms were impaired work performance 325 (55%), lack of interest in usual activities 315 (53.3%) and obsessional thoughts 303 (51.3%).The data showed that the number of premenstrual symptoms experienced by the students varied from one individual to the other. The average number of premenstrual symptoms experienced by the students was 20.8. The majority 314 (53.0%) of the respondents experienced more than half of the symptoms (see Additional File 3).

The degree of severity of the premenstrual symptoms in students was investigated based on an index, which measured the number symptom (indicator) occurred and rating severity of symptoms. All the indicators were then added together to form the index. The index scores ranged between 40 indicating that there were no symptoms to 200 indicating that the symptoms were very high or at the maximum severity. The mean number of symptoms experienced by students was 20.8 ± 9 standard deviation and a mode of 29 and a median of 21.

**Fig. 3:**
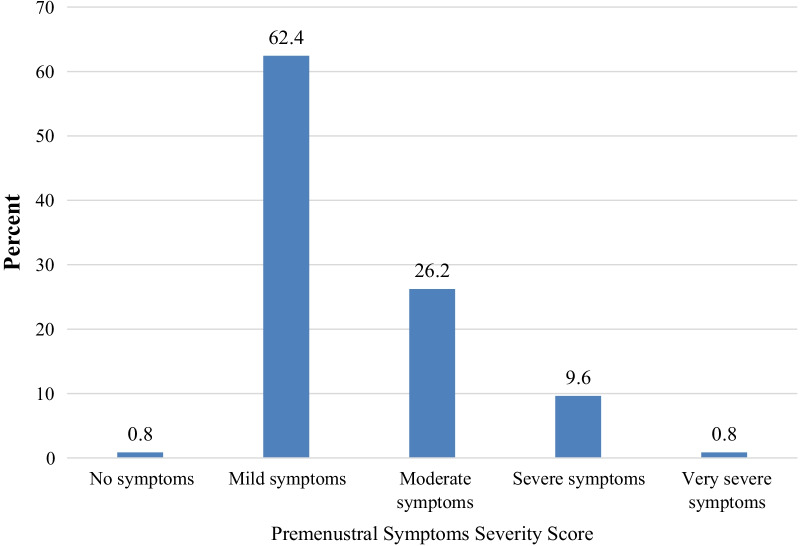
Premenstrual Symptoms Severity Score of study participants, Wolkite University, Ethiopia, 2021

The majority (62.4%) of the students had mild level of severity of the symptoms and 0.8% had none/minimal levels of severity of the symptoms (Fig. 3).

### Magnitude of premenstrual syndrome

**Fig. 4:**
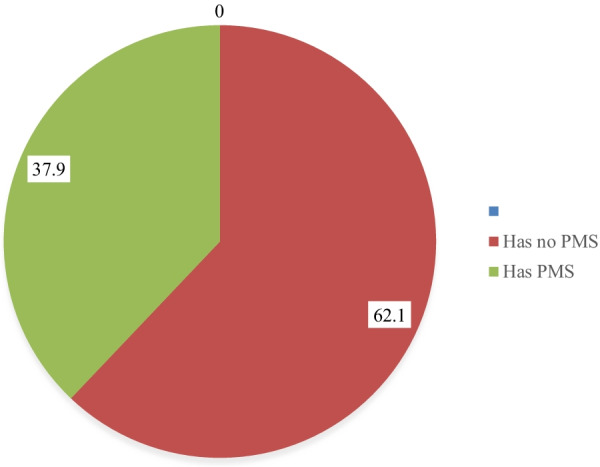
Magnitude of premenstrual syndrome among Wolkite University regular female students, Ethiopia, 2021

From 591 study participants 224 (37.9%) [95% CI: (34, 40.9)] of them had premenstrual syndrome, of those 103 (56.6%) had family history of PMS and 22 (42.3%) of them have body mass index (BMI) > 24.9 kg/m^2^ (Fig. 4).

### Coping mechanisms for premenstrual symptoms

From a total study participants 552 (93.4%) of them apply at least one coping mechanism for premenstrual symptoms regardless of its severity. From coping mechanisms, taking rest accounts for 373 (67.6%) of the study participants followed by sleeping 335 (60.7%) (Table 3).

**Table 3:** Coping mechanisms of the study participants for premenstrual symptoms, Wolkite University, Ethiopia, 2021

Coping mechanisms	Frequency	Percentage (%)
Taking rest	373	67.6
Sleeping	335	60.7
Applying hot packs	163	29.5
Taking anti-pain drugs	155	28.1
Body massage	149	26.9
Listening music	136	24.6
Taking hot shower	117	21.2
Caffeine intake	112	20.3
Diet alteration	102	18.5
Crying	80	14.5
Go to clinic	75	13.6
Doing exercise	65	11.8
Taking herbal (traditional) drugs	27	4.9
Drinking alcohol	16	2.9
Smoking cigarette	7	1.3
Others (drinking Coca-Cola, warm compress at the abdomen, drinking hot water and make one self-busy on other activities	12	2.2

### Factors associated with premenstrual syndrome

Both bi-variable and multi-variable binary logistic regression analysis was computed and those variables with *P *value of less than 0.05 in bi-variable logistic regression analysis were included in the final model. In bivariate analysis factors such as: Family marital status, educational level of the mother, perceived income status of the family, family size, family history of PMS, habit of physical exercise, history of sexual intercourse, milk consumption, information about PMS, age at menarche, duration of menses, perceived menstrual pain intensity, menstrual interval, amount of pads used during menstruation, amount of menstrual bleeding, menstrual regularity and use of any hormonal contraceptives were significantly associated with PMS. In multi-variable analysis; Family history of PMS, history of sexual intercourse, perceived menstrual pain intensity, amount of pads used during menstruation, menstrual regularity, age at menarche, duration of menses were factors significantly associated with premenstrual syndrome at *P *value < 0.05 with 95% confidence interval.

Those students having family history of PMS were 4.05 [95% CI: (2.49, 6.58)] times more likely to develop premenstrual syndrome as compared to those without family history. Students with history of sexual intercourse were almost 2 [95% CI: (1.12, 3.47) times less likely to develop PMS in comparison with the counterpart. The odds of having premenstrual syndrome for those with moderate and severe perceived menstrual pain intensity were 1.84 [95% CI: (1.02, 3.30)] and 3.09 [95% CI: (1.58, 6.05)] respectively. Students with irregular menstrual cycle were 2.26 [95% CI: (1.41, 3.62)] times more risky to experience premenstrual syndrome than the counterpart and those who start menstruation at early age (< 13 years) are 2.64 [95% CI: (1.34, 5.19)] times more likely to develop PMS. Those students having long menses (≥ 7 days) were 3.56 [95% CI: (1.53, 8.37)] times more likely to have PMS than those with menstrual duration of 1–3 days. Finally, this study indicates that those who used 4–8 pads and > 8 pads during menstruation were 2.42 [95% CI: (1.31, 4.46)] and 4.44 [95% CI: (2.16, 9.12)] times more to develop PMS respectively as compared to students who used 1–3 pads (Table 4).

**Table 4:** Multivariable binary logistic regression to identify factors associated with premenstrual syndrome, Wolkite University, Ethiopia, 2021

Explanatory variable	Category	Premenstrual Syndrome		COR (95% CI)	AOR (95% CI)	*P *value
		YES	NO			
Perceived familial history of premenstrual syndrome	Yes	103	79	1.00	1.00	
	No	87	254	0.26 (0.18, 0.39)*	0.25 (0.15, 0.40)*	0.001*
	Do not know	34	34	0.77 (0.44, 1.34)	1.02 (0.51, 2.05)	0.953
History of sexual intercourse	Yes	50	134	0.50 (0.34, 0.73)*	0.51 (0.29, 0.89)*	0.019*
	No	174	233	1.00	1.00	
Age at menarche	< 13 years	34	31	1.94 (1.16, 3.26)*	2.64 (1.34, 5.19)*	0.005*
	> = 13 years	190	336	1.00	1.00	
Duration of menses	1–3 days	41	96	1.00	1.00	
	4–6 days	148	248	1.39 (0.92, 2.12)	1.04 (0.61, 1.78)	0.889
	> = 7 days	35	23	3.56 (1.88, 6.76)*	3.58 (1.53, 8.37) *	0.003*
Menstrual pain intensity level	Mild	33	112	1.00	1.00	
	Moderate	109	204	1.81 (1.15, 2.85)*	1.84 (1.02, 3.30)*	0.043*
	Severe	82	51	5.46 (3.24, 9.20)*	3.09 (1.58, 6.05)*	0.001*
Number of pads (modes) used during menstruation	< 4 pads	29	87	1.00	1.00	
	4–8 pads	108	218	1.49 (0.92, 2.40)	2.42 (1.31, 4.46)*	0.005*
	> 8 pads	87	62	4.21 (2.47, 7.16)*	4.44 (2.16, 9.12)*	0.001*
Does your menstrual cycle regular?	Yes	123	271	0.43 (0.30, 0.61)*	0.44 (0.28, 0.71)*	0.001*
	No	101	96	1.00	1.00	

‘’*’’for variables significantly associated with premenstrual syndrome at *P *value < 0.05; Bold used to easily identification of significantly associated variables

## Discussion

This study revealed evidence on premenstrual syndrome by determining its magnitude, severity, coping mechanisms and identifying risk factors of the syndrome among Wolkite Uviversity female students. In this study the magnitude of premenstrual syndrome was 37.9% [95% CI: (34, 40.9)], in line with a study conducted in Mekelle University, Ethiopia (37.0%) [4]. This implies premenstrual syndrome is a common problem of female University students, indicating a need to give emphasis for it by policy makers and health care providers. This finding was also similar with a study conducted in Saudi Arabia (35.6%) [14]. This might be due to similar in study setting in which both were institutional based studies, similarity in tool used to determine PMS and study population. On the other hand, this finding was higher than studies done among college students of Bhavnagar, Gujarat (18.4%) [15], Iran (30.7%) [10], Thailand (20.5%) [22], Thai (16.8%) [16] and Ambo University, Ethiopia (18%) [26]. The discrepancy from Ambo University, Ethiopia might be due to difference in instrument used to assess PMS, in which a study in Ambo University use 19-item containing tool, whereas, the current study used 40 items containing instrument. Others were community based studies involving all age groups in contrast to this study which was conducted among university students with stressful life potentiating psychological symptoms, which increases its prevalence in the current study. To the opposite this finding was lower than a study conducted in Switzerland (81%) [13], Turkey (71.3%) [24], Ordu, in the East Black Sea region of Turkey (49.7%) [7], Iran (85.6%) [9], Bahir Dar University (72.8%) [1], Debremarkos town (81.3%) [8] and Addis Ababa, Ethiopia (86.1%) [17]. The discrepancies from study conducted in Ethiopia at Bahir Dar University might be due to difference in tool used to determine presence of menstrual syndrome. Its discrepancy from studies conducted in Debremarkos town and Addis Ababa, Ethiopia might be difference in age of the participants and study setting where others were community based studies involving all reproductive age women’s. In addition to this, difference in socio-economic status, study setting and genetic difference from other countries might be the reason.

This study revealed that, students having family history of PMS were 4.05 times more likely to develop premenstrual syndrome as compared to those without family history. This implies that having family history of PMS had a positive impact on the presence of PMS. This finding was in line with studies conducted in Bahidar University [1], Ordu [7] and Saudi Arabia [14]. This indicates that PMS has genetic predisposition (genetic factor), and might be due to shared biological and/or psychological factors which may influence expectations and self-awareness, genetic similarity in cellular sensitivity and responsiveness to estrogen and progesterone hormone [5]. This indicates a need for mothers with premenstrual syndrome to discussion with their daughter on coping mechanisms and psychological readiness to decrease its impact. Students with history of sexual intercourse were almost 2 times less likely to develop PMS in comparison with the counterpart. This showed that being married can be preventive measure for PMS, which might be due to stimulation of parasympathetic nervous system during sexual act decreases pain perception and release of happiness hormone and sense of affection during sexual intercourse provide psychological comfort. These, being married can be considered as one of the options that can be used as a preventive measure. Similarly in this study, the odds of having premenstrual syndrome for those with moderate and severe perceived menstrual pain intensity were 1.84 and 3.09 respectively, consistent with a study conducted in Ordu [7], Turkey. This might be due to physiologic effect of pain; severe pain may cause loss of appetite, anxiety, mood disturbance, loss of concentration, work impairment and feeling of guilty for being female that predispose them to develop psychological symptoms and behavioral change leading to premenstrual syndrome [5]. This implies the need for seeking health care and applying of non-pharmacological pain management strategies for students with severe menstrual pain to reduce occurrences of associated symptoms, all in sum leading to premenstrual syndrome.

Students with irregular menstrual cycle were 2.26 times more risky to experience premenstrual syndrome than the counterpart. This finding was similar with studies conducted in Bahir dar [1], Ordu and Saudi Arabia [14]. The possible justification for this might be due irregularity of menstruation could fluctuate steroid hormones and unexpected occurrence of menses irregularly without mental adjustment leads to psychological disturbance and associated behavioral change. Those students who start menstruation at early age (< 13 years) were 2.64 times more likely to develop PMS, consistent with a study conducted in Saudi Arabia [14] and Debre Markos [8]. The findings in this study could be explained with the fact that earlier age of menarche are associated with early establishment of ovarian functions and ovulation with fluctuation of steroid hormones in such a young age with less physical and psychological maturity which may lead to PMS manifestations [27]. Those findings indicates that prompt identification and management of menstrual irregularity and providing counseling service for those who started menstruation at early age is crucial to decrease severity of premenstrual symptoms.

In this study those students having long menses (≥ 7 days) and using many pads during menstruation were more likely to have PMS, similar with studies conducted in Mekelle University [4]. This association could be due to repeated class absenteeism, prolonged hormonal effect, decreased sense of body cleanliness, disturbed study time and associated poor academic performance, all leading to PMS [9]. Heavy menstrual blood flow not only causes psychological disturbances but also pose a risk for acute complications and chronic diseases that it needs great attention by students, parents and health care providers.

## Conclusions

In this study premenstrual syndrome was found to be a problem of many students. Abdominal cramp, depressed feeling and fatigue were the predominant premenstrual symptoms experienced by students. Taking rest and sleeping were mostly applied by students as a coping mechanism. Family history of PMS, no history of sexual intercourse, intense menstrual pain, using of many pads during menstruation, irregular menstrual cycle, early menarche, and long duration of menses were found to be predictors’ of premenstrual syndrome. This study indicates the need for policy makers to giving emphasis for it by considering PMS as a component of routine health care for women’s and health care professionals should be aware of the problem and provide comprehensive service for university students. The university must also create awareness for students about the problem and effective coping mechanisms. Parents should openly discuss with their female students on the issue to have psychological stability. Finally it is recommended for interested researchers to conduct study on this issue including effectiveness of coping strategies using prospective cohort study design with diary record of symptoms to avoid recall bias.

## Limitations of the study

It was a cross-sectional study that might not show cause and effect relationship and didn’t assess degree of effectiveness of coping mechanisms. It might also pose recall bias for some of past events like age of menarche and experienced premenstrual symptoms.

## Supplementary Information

**Additional file 1.** Schematic presentation of sampling procedure to select study participants, Wolkite University, Wolkite, Ethiopia, 2021.

**Additional file 2.** Socio-demographic characteristics of the study participants, Wolkite University, Wolkite, Ethiopia, 2021.

**Additional file 3.** Frequency of premenstrual symptoms of the study participants, Wolkite University, Ethiopia, 2021.

## Data Availability

The datasets used and/or analyzed during the current study will be available from the corresponding author on reasonable request.

## Abbreviations

AOR: Adjusted odds ratio
ACOG: American college of obstetrics and gynecology
PMS: Premenstrual syndrome
PMDD: Premenstrual disphoric disorder
PMSS: Premenstrual syndrome scale
SPSS: Statistical package for social science
WHO: World Health Organization
