# Supplementary material for: Premenstrual syndrome, coping mechanisms and associated factors among Wolkite university female regular students, Ethiopia, 2021
Source: BMC Womens Health. 2022 Mar 23;22:88. doi: 10.1186/s12905-022-01658-5 (PMC8942154; doi:10.1186/s12905-022-01658-5)
Supplement: Supplementary file 1 — Additional file 1. Schematic presentation of sampling procedure to select study participants, Wolkite University, Wolkite, Ethiopia, 2021. [file 12905_2022_1658_MOESM1_ESM.docx]

**Wolkite University**

Engineering college (505)

School of law (96)

Computer Science College (170)

Behavioral Science College (114)

Social Science College (147)

Agriculture College (145)

Natural and computational Science College (172)

Business and Economics College (145)

Medicine and health Science College (210)

Engineering college

505*(631/885) = **360**

Medicine and health Science College

210*(631/885) = **150**

Computer Science College

170*(631/885) = **121**

**Additional files 1:** Schematic presentation of sampling procedure to select study participants, Wolkite University, Wolkite, Ethiopia, 2021
