# Supplementary material for: Premenstrual syndrome, coping mechanisms and associated factors among Wolkite university female regular students, Ethiopia, 2021
Source: BMC Womens Health. 2022 Mar 23;22:88. doi: 10.1186/s12905-022-01658-5 (PMC8942154; doi:10.1186/s12905-022-01658-5)
Supplement: Supplementary file 2 — Additional file 2. Socio-demographic characteristics of the study participants, Wolkite University, Wolkite, Ethiopia, 2021. [file 12905_2022_1658_MOESM2_ESM.docx]

Additional file 2: Socio-demographic characteristics of the study participants, Wolkite University, Wolkite, Ethiopia, 2021

| Socio-demographic variable | Category | Frequency | Percentage (%) |
| --- | --- | --- | --- |
| Age | <20 years | 53 | 9% |
|  | >20 years | 538 | 91% |
| Marital status | Single | 429 | 72.6% |
|  | Married | 139 | 23.5% |
|  | Widowed | 19 | 3.2% |
|  | Divorced | 4 | 0.7% |
| Religion | Orthodox | 334 | 56.5% |
|  | Muslim | 129 | 21.8% |
|  | Protestant | 100 | 16.9% |
|  | Catholic | 28 | 4.7% |
| Ethnicity | Amhara | 212 | 35.9% |
|  | Oromo | 124 | 21.0% |
|  | SNNPR | 188 | 31.8% |
|  | Tigray | 49 | 8.3% |
|  | Others | 18 | 3.0% |
| Residency | Urban | 429 | 72.6 |
|  | Rural | 162 | 27.4 |
| College | Engineering | 341 | 57.7 |
|  | Medicine and health science | 137 | 23.2 |
|  | Computer science | 113 | 19.1 |
| Family size | 1-3 | 46 | 7.8 |
|  | 4-6 | 357 | 60.4 |
|  | >7 | 188 | 31.8 |
| Family marital status | Family live together | 495 | 83.8 |
|  | Divorced family | 43 | 7.3 |
|  | Extended family | 28 | 4.7 |
|  | Others | 25 | 4.2 |
| Mother educational status | Cannot read and write | 157 | 26.6 |
|  | Primary school | 189 | 31.9 |
|  | Secondary school | 114 | 19.3 |
|  | College and above | 131 | 22.2 |
| Perceived family income | Adequate | 268 | 45.3 |
|  | Partially adequate | 212 | 35.9 |
|  | Higher | 69 | 11.7 |
|  | Inadequate | 42 | 7.1 |
| Family history of PMS | No | 341 | 57.7 |
|  | Yes | 182 | 30.8 |
|  | Do not know | 68 | 11.5 |
