# Supplementary material for: Premenstrual syndrome, coping mechanisms and associated factors among Wolkite university female regular students, Ethiopia, 2021
Source: BMC Womens Health. 2022 Mar 23;22:88. doi: 10.1186/s12905-022-01658-5 (PMC8942154; doi:10.1186/s12905-022-01658-5)
Supplement: Supplementary file 3 — Additional file 3. Frequency of premenstrual symptoms of the study participants, Wolkite University, Ethiopia, 2021. [file 12905_2022_1658_MOESM3_ESM.docx]

Additional file 3: Frequency of premenstrual symptoms of the study participants, Wolkite University, Ethiopia, 2021

| **Variables** | **Frequency [N=591]** | **Percent (%)** |
| --- | --- | --- |
| **Physiological symptoms** |  |  |
| Abdominal cramps | 466 | 78.8 |
| Fatigue | 431 | 72.9 |
| Headache | 388 | 65.7 |
| Muscle and Joint pain | 357 | 60.4 |
| Generalized aches and pains | 351 | 59.4 |
| Abdominal bloating | 345 | 58.4 |
| Breast tenderness and swelling | 307 | 51.9 |
| Skin color changes, rashes, pimples | 304 | 51.4 |
| Nausea/vomiting | 298 | 50.4 |
| Palpitations | 274 | 46.4 |
| Dizziness/fainting | 249 | 42.1 |
| Change in bowel habits | 247 | 41.8 |
| Increased appetite | 247 | 41.8 |
| Pelvic discomfort and pain | 236 | 39.9 |
| Weight gain | 225 | 38.1 |
| Food cravings (Sugar/ Salt) | 220 | 37.2 |
| **Psychological symptoms** |  |  |
| Depression | 433 | 73.3 |
| Mood swings | 415 | 70.2 |
| Irritability | 403 | 68.2 |
| Aggression | 380 | 64.3 |
| Tension | 364 | 61.6 |
| Sleep changes (Insomnia/ hypersomnia) | 346 | 58.5 |
| Loss of concentration | 342 | 57.9 |
| Confusion | 323 | 54.7 |
| Forgetfulness | 310 | 52.5 |
| Easy crying/ Crying spells | 309 | 52.3 |
| Anxiety | 309 | 52.3 |
| Hopelessness | 278 | 47.0 |
| **Behavioral symptoms** |  |  |
| Impaired work performance | 325 | 55.0 |
| Lack of interest in usual activities | 315 | 53.3 |
| Obsessional thoughts | 303 | 51.3 |
| Poor judgment | 294 | 49.7 |
| Restlessness | 290 | 49.1 |
| Irrational thoughts | 256 | 43.3 |
| Lack of self-control | 242 | 40.9 |
| Compulsive behavior | 239 | 40.4 |
| Social withdrawal | 232 | 39.3 |
| Being over sensitive | 227 | 38.4 |
| Feeling guilty | 210 | 35.5 |
| Clumsiness | 184 | 31.1 |
